# Déficit congénital en facteur V: à propos d’un cas

**DOI:** 10.11604/pamj.2017.27.182.12285

**Published:** 2017-07-06

**Authors:** Saloua Boujrad, Brahim El Hasbaoui, Hanae Echahdi, Mohamed Malih, Aomar Agadr

**Affiliations:** 1 Service de Pédiatrie, Hôpital Militaire d’Instruction Mohamed V, Faculté de Médecine et de Pharmacie, Université Mohammed V, Rabat, Maroc

**Keywords:** Déficit congénital en facteur V, épistaxis, circoncision, plasma frais congelé, Factor V congenital deficiency, epistaxis, circumcision, fresh frozen plasma

## Abstract

Le déficit congénital en facteur V est une anomalie rare de la coagulation, initialement décrite par Owren en 1947 et connue sous le nom de para hémophilie. Elle est transmise selon un mode autosomique récessif. En générale elle est symptomatique à l'état homozygote. Le facteur V est un cofacteur essentiel dans la conversion de la prothrombine en thrombine par le facteur X activé. En l'absence du facteur V, la génération de thrombine est ralentie et la formation de fibrine est retardée. Il en résulte une tendance aux saignements. Nous rapportons un cas de déficit congénital en facteur V chez un nourrisson présentant des épistaxis à répétition.

## Abstract (en)

Factor V congenital deficiency is a rare coagulation disorder initially described by Owren in 1947 and known as para hemophilia. It is transmitted through autosomal-recessive inheritance and homozygous cases are usually symptomatic. Factor V is an essential cofactor in the conversion of prothrombin to thrombin by activated factor X. In the absence of factor V, thrombin generation is slowed down and fibrin formation is delayed. This results in a bleeding tendency. We report a case of factor V congenital deficiency in an infant with recurrent epistaxis.

## Introduction

Le déficit congénital en facteur V est une anomalie rare de la coagulation, initialement décrite par Owren en 1947 et connue sous le nom de para hémophilie. Elle est transmise selon un mode autosomique récessif. En générale elle est symptomatique à l'état homozygote [1]. Le facteur V est un cofacteur essentiel dans la conversion de la prothrombine en thrombine par le facteur X activé. En l'absence du facteur V, la génération de thrombine est ralentie et la formation de fibrine est retardée. Il en résulte une tendance aux saignements [2]. Le déficit en facteur V est une coagulopathie congénitale rare, sa prévalence est estimée à une personne pour un million d'habitants [3, 4], se manifestant à tout âge par un syndrome hémorragique de sévérité variable, dont le diagnostic repose sur le bilan d'hémostase avec dosage du facteur V et dont le traitement se base sur la perfusion de PFC [5].

## Patient et observation

Nous rapportons le cas d'un nourrisson de neuf mois, de sexe masculin unique de sa famille issu d'un mariage consanguin, non circoncis ayant comme antécédents, notion de céphalhématome à la naissance qui s'est résorbé spontanément deux mois plus tard. Le nourrisson était vu en consultation pour des épistaxis à répétition depuis la naissance avec accentuation au fil du temps, mais aucune investigation d'hémostase n'avait été réalisée. Le bilan d'hémostase révélait un TP bas à 12%, un temps céphaline Kaolin (TCK) hors plage de mesure et un taux de facteur V effondré à 2% avec des taux normaux des autres facteurs de la coagulation. L´enquête familiale a révélé l'existence chez les deux parents d'un déficit hétérozygote avec des taux de facteur V à 44% et à 59%. Le diagnostic d'un déficit congénital en facteur V était alors retenu. Vu le risque hémorragique, un traitement substitutif était décidé. Ce dernier était à base de PFC (plasma frais congelé) à raison de 20ml/kg. Le TP de contrôle après transfusion était de 70% avec arrêt du saignement au décours de cette perfusion. Une circoncision a été programmée chez ce nourrisson. L'apport du PFC était poursuivi en période postopératoire à raison de 20ml/kg par six heures pendant les premières 24 heures, puis 10ml/kg par 12 heures en trois prises pendant les six jours postopératoires suivants jusqu'à la chute de l'escarre. Aucun incident postopératoire hémorragique ou thrombotique n'était noté, l'enfant était déclaré sortant du service une semaine après l'intervention.

## Discussion

Le déficit en facteur V représente un désordre rare de la coagulation avec une incidence de un pour 10000 [6]. Il peut être isolé ou associé à un déficit congénital en facteur VIII. Il est dû à des mutations du gène F5 (1q23) qui contrôle la production du facteur V plasmatique. La transmission est autosomale récessive. Les manifestations cliniques sont variables et les saignements sont parfois mineurs voire absents [7]. Le plus souvent, il s'agit d'ecchymoses, d'épistaxis, de méno-métrorragies chez les jeunes filles et les femmes [8] ou d'hémorragies après un acte invasif (circoncision [9] ou une extraction dentaire), post-traumatiques ou post-opératoires. Plus rarement, des hématomes profonds et des hémarthroses sont observés. Les hémorragies cérébrales anté- ou postnatales sont exceptionnelles [6]. Les formes les plus sévères se manifestent tôt dans l'enfance [3]. Aucune prédisposition ethnique n'a été rapportée [10]. Le déficit en facteur V est 10 fois plus fréquent dans les pays à consanguinité élevée [11]. Plus de 200 cas ont été rapportés dans la littérature [12]. II n'y aurait pas de relation entre le taux plasmatique de facteur V et la sévérité des saignements qui seraient plus en rapport avec le taux plaquettaire de facteur V [13]. Le diagnostic se base sur l´allongement du temps de Quick et du temps de céphaline activée et d´une diminution du taux de facteur V dosé par la thromboplastine. L´analyse moléculaire est possible mais n´est pas nécessaire pour le diagnostic. Le diagnostic différentiel inclut le déficit en facteur VIII et le déficit combiné en facteurs V et VIII [6]. Les concentrés en FV ne sont pas disponibles, le plasma frais congelé (PFC) est donc le seul traitement disponible s´il faut corriger le déficit [14]. Le traitement peut être administré une fois par jour pendant sept jours au moment d'une intervention chirurgicale et probablement pas plus d'une fois pour un saignement mineur, dans des cas extrêmes d´hémorragie sévère, la transfusion de concentrés plaquettaires peut s´avérer utile en complément du PFC [3–15]. Fratantoni et al [16] rapportent le cas d'une fille porteuse d'un déficit congénital de facteur V qui a développé un anticoagulant circulant contre le facteur V transfusé comme complication de la transfusion. Le pronostic est favorable lorsque la maladie est diagnostiquée et traitée de manière adéquate.

## Conclusion

Le déficit congénital en facteur V est une anomalie rare de la coagulation. Elle est transmise selon le mode autosomique récessif et est symptomatique à l'état homozygote. Il en résulte une tendance aux saignements. L'intervention chirurgicale sur ce terrain nécessite une préparation au préalable par perfusion de plasma frais congelé suivant un protocole bien codifié.

## Conflits d’intérêts

Les auteurs ne déclarent aucun conflit d'intérêt.

## References

[1] Owren PA (1947). The coagulation of Blood: investigations on a new clotting factor. Acta Med Scand..

[2] Seeler RA (1972). Parahemophilia: factor V deficiency. Med Clin North Am..

[3] Huang JN, Koerper MA (2008). Factor V deficiency: a concise review. Haemophilia..

[4] Sanklecha MU, Sundaresan S, Charde V (2014). Factor V Deficiency: a Subtle Presentation. Indian J Pediatr..

[5] Benjelloun O (2010). Déficit congénital en facteur V dans une fratrie. Archives de Pédiatrie..

[6] Vigue B, Treamey B, Tazarourte K (2007). Hémorragies sous antivitamines K : les freins à la prescription du PPSB, mythes et réalités. JEUR..

[7] Bounes V, Lagarde F, Battefort F, Ruols E, Frontin P, Ducassé JL (2008). Impact de l'échographie préhospitalière lors de son utilisation en routine. JEUR..

[8] Chemaou A, Ayachi A, Benjelloun O, Zineddine A (2013). Ménorragies par déficit congénital en facteur V chez une adolescente. Archives de Pédiatrie..

[9] El Koraichi A, Mokhtari M, Ghannam M, Mekaoui N, El Haddoury M, Ech-Cherif El Kettani S (2011). Déficit en facteur V et circoncision : gestion périopératoire : à propos d'un cas clinique. Ann Fr Anesth Reanim..

[10] Kalafatis M (2005). Coagulation factor V: a plethora of anticoagulant molecules. Curr Opin Hematol..

[11] Lak M, Sharifian R, Peyvandi F (1998). Symptoms of inherited factor V deficiency in 35 Iranian patients. Br J Haematol..

[12] Asselta R, Peyvandi F (2009). Factor V deficiency. Semin Thromb Hemost..

[13] Song JW, Um MR, Ahn HS, Hong CY (1987). A case of congenital factor V deficiency. J Korean Med Sci..

[14] consulte EM (2009). L'hémostase en pédiatrie et ses principales pathologies. OptionBio..

[15] Thalji N, Camire RM (2013). Parahemophilia: new insights into factor v deficiency. Semin Thromb Hemost..

[16] Fratantoni JC, Hilgartner M, Nachman RL (1972). Nature of the defect in congenital factor V deficiency: study in a patient with an acquired circulating anticoagulant. Blood..

